# Ebola Virus Neutralizing Antibodies in Dogs from Sierra Leone, 2017

**DOI:** 10.3201/eid2604.190802

**Published:** 2020-04

**Authors:** Kerstin Fischer, Roland Suluku, Sarah Katharina Fehling, Juliet Jabaty, Bashiru Koroma, Thomas Strecker, Martin H. Groschup, Sandra Diederich

**Affiliations:** Friedrich-Loeffler-Institut, Greifswald-Insel Riems, Germany (K. Fischer, M.H. Groschup, S. Diederich);; Njala University, Bo, Sierra Leone (R. Suluku, B. Koroma);; Philipps University of Marburg, Marburg, Germany (S.K. Fehling, T. Strecker);; Sierra Leone Agricultural Research Institute, Freetown, Sierra Leone (J. Jabaty)

**Keywords:** Ebola virus, dogs, Sierra Leone, neutralizing antibodies, serology, viruses, zoonoses

## Abstract

Ebola virus (EBOV) is a highly pathogenic zoonotic virus for which the reservoir host has not been identified. To study the role of dogs as potential hosts, we screened 300 serum samples from dogs in Sierra Leone and found EBOV neutralizing antibodies in 12, suggesting their susceptibility to natural infection.

Ebolaviruses (family *Filoviridae*) comprise highly pathogenic RNA viruses with zoonotic potential. After sporadic introduction from an animal reservoir into the human population, the main route of transmission has been from human to human, causing outbreaks of hemorrhagic fever with case-fatality rates up to 90% (*1*). Although molecular and serologic evidence strongly points toward certain species of bat as reservoir hosts for ebolaviruses (*2*,*3*), a bat-derived Ebola virus (EBOV) isolate has not yet been detected. Despite intensive serologic surveillance focusing on the role of bats, wildlife, and livestock in EBOV ecology (*2*,*4*–*7*), to our knowledge, only 2 reports describe analysis of serum from dogs in Gabon and Liberia after Ebola virus disease (EVD) outbreaks in 2001 (Gabon) and 2014–2016 (Liberia) (*8*,*9*). Although antibodies against EBOV were detected by indirect ELISA, neither EBOV antigen nor viral genome was detected in samples from Gabon. The highest seroprevalence (31.8%) was reported from villages where dogs were reportedly exposed to the virus through contact with human EVD patients or by eating infected animal carcasses (*8*). In Liberia, a multiplex approach indicated that 47 (73%) of 64 dogs had potentially been exposed to filoviruses (*9*). To further investigate the role of dogs in EBOV ecology, we collected 300 serum samples from 174 male (58%) and 126 female (42%) dogs in Moyamba District, Sierra Leone (Figure 1). 

**Figure 1:**
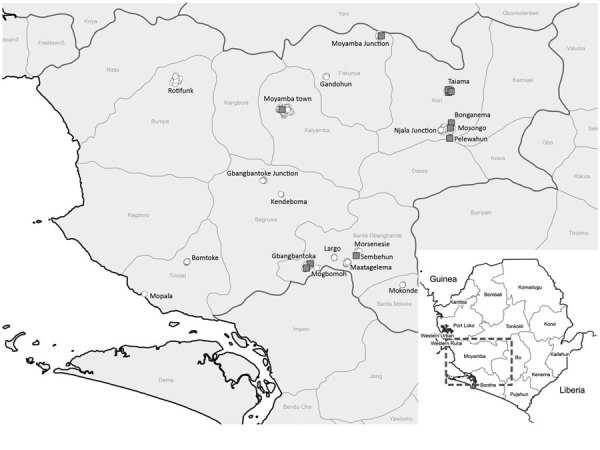
Sampling locations for study of Ebola virus neutralizing antibodies in dogs, Moyamba District, Sierra Leone, 2017. White circles indicate sampling locations; gray squares indicate dog serum samples with virus neutralizing activity. Inset shows location of Moyamba district in Sierra Leone.

## The Study

We sampled only owned and healthy dogs from communities that have been affected by the West Africa EVD outbreak (Appendix). Sampling was performed in rural and urban areas that had been affected by the historically largest EVD outbreak involving the Makona strain during 2014–2016 in West Africa. At the time of sample collection (October–December 2017), of the 300 dogs, 163 were >2 years of age (Table 1). Animals were handled according to a Njala University Institutional Review Board protocol (no. IRB00008861/FWA00018924).

**Table 1:** EBOV-specific antibodies detected in dog serum samples, by dog age, collected in Moyamba District, Sierra Leone, October–December 2017*

Age, mo.	No. samples tested	EBOV-NP ELISA, no. (%) reactive	Confirmatory EBOV-NP WB, no. (%) reactive	EBOV VNT	
No. (%) positive	Titers
<12	27	2 (7.4)	0	0	NA
12 –18	60	7 (11.7)	6 (10.0)	5 (8.3)	1:11, 1:11, 1:13, 1:16, 1:27
19–24	50	4 (8.0)	3 (6.0)	1 (2.0)	1:11
25–36	90	11 (12.2)	5 (5.6)	4 (4.4)	1:16, 1:16, 1:19, 1:32
37–48	39	7 (17.9)	5 (12.8)	5 (12.8)	1:11, 1:16, 1:19, 1:23,1:45
>48	34	5 (14.7)	1 (2.9)	3 (8.8)	1:11, 1:19, 1:19
Total	300	36 (12.0)	20 (6.7)	18 (6.0)	

*EBOV, Ebola virus; NA, not applicable; NP, nucleoprotein; VNT, virus neutralization test; WB, Western blot.

**Figure 2:**
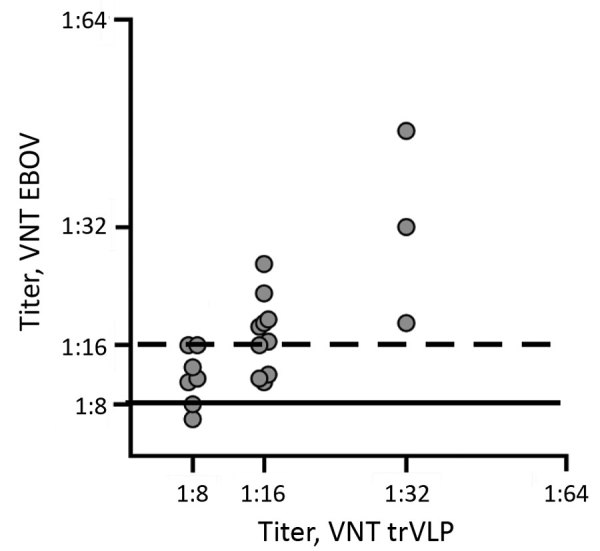
Analysis of dog serum samples (circles) in VNTs for study of EBOV neutralizing antibodies in dogs, Moyamba District, Sierra Leone, 2017. Comparison of dog serum titers obtained in VNTs was based on live EBOV (variant Mayinga) and EBOV trVLP. For VNT using authentic EBOV, serum samples with a titer <1:8 (horizontal solid line) are counted as negative; samples with a neutralizing titer >1:8 are considered positive. For trVLP-based VNT, titers equal to 1:16 (horizontal dashed line) are counted as positive. EBOV, Ebola virus; trVLP, transcription and replication competent virus-like particles; VNTs, virus neutralization tests.

Initially, we screened dog serum samples for the presence of EBOV nucleoprotein (NP)–specific antibodies in an indirect ELISA, as previously described for pigs (*5*), with slight modifications. Using a horseradish peroxidase–labeled protein A/G–specific conjugate, we considered 36 (12%) serum samples to be reactive toward the *Escherichia coli*–derived EBOV-NP (Tables 1, 2). Subsequent Western blot analyses based on insect cell–derived EBOV-NP (*5*) confirmed the presence of EBOV-NP reactive antibodies in 20 (6.6%) samples. Furthermore, we performed virus neutralization tests (VNTs) by using transcription and replication competent virus-like particles (trVLP) and authentic EBOV (variant Mayinga) as described previously (*5**,**10*) (Appendix). We found that 12 (4%) serum samples efficiently inhibited EBOV infection with robust neutralizing titers of 1:16–1:45 and that another 6 samples had weakly positive titers of 1:11–1:13 (Tables 1, 2). Overall, titers from the trVLP-based VNTs with an established cutoff at 80% inhibition of reporter activity were comparable to those of VNTs with live virus (Figure 2).

**Table 2:** EBOV-specific antibodies detected in dog serum samples, according to sampling region, Sierra Leone, October–December 2017*

Region	No. samples tested	EBOV-NP ELISA, no. (%) reactive	Confirmatory EBOV-NP WB, no. (%) reactive	EBOV VNT	
				No. (%) positive	Titers
Bomtoke	11	0	0	0	NA
Bonganema	8	3 (37.5)	3 (37.5)	1 (12.5)	1:16
Gandohun	4	0	0	0	NA
Gbangbantoke	24	3 (12.5)	0	3 (12.5)	1:13, 1:19, 1:19
Gbangbantoke Junction	14	0	0	0	NA
Kendeboma	7	0	0	0	NA
Largo	9	0	0	0	NA
Matagelema	16	1 (6.3)	1 (6.3)	0	NA
Mogbomoh	4	0	0	0	NA
Mokonde	14	1 (7.1)	1 (7.1)	0	NA
Mopala	1	0	0	0	NA
Morsenesie	4	2 (50.0)	0	0	NA
Mosongo	26	5 (19.2)	3 (11.5)	2 (7.6)	1:11; 1:16
Moyamba Junction	16	2 (12.5)	2 (12.5)	1 (6.2)	1:23
Moyamba Town	62	1 (1.6)	1 (1.6)	1 (1.6)	1:32
Njala Junction	15	2 (13.3)	1 (6.6)	1 (6.6)	1:11
Pelewahun	14	2 (14.3)	2 (14.3)	1 (7.1)	1:45
Rotifunk	21	2 (9.5)	2 (9.5)	2 (9.5)	1:11, 1:11
Sembehun	7	3 (42.9)	1 (14.3)	1 (14.2)	1:27
Taiama	23	9 (39.1)	3 (13.0)	5 (21.7)	1:11, 1:16, 1.16, 1:19, 1.19
Total	300	36 (12.0)	20 (6.7)	18 (6.0)	

*EBOV, Ebola virus; NA, not applicable; NP, nucleoprotein; VNT, virus neutralization test; WB, Western blot.

## Conclusions

EBOV seroprevalence detected by ELISA in dogs from EVD-endemic areas in Gabon (25.2%–31.8%) (*8*) was lower than that detected in dogs in our study in Sierra Leone (12%). Technically, differences in detectable seroprevalence could be explained by different preparations used (virus-infected cell lysates in ELISA in Gabon [*8*] compared with single EBOV-NP preparations in our study). Apart from that difference, the observed variation might depend on selected sampling areas, animals, and time points of sampling because stability and persistence of neutralizing and NP-reactive antibodies in dogs after exposure are unknown. In pigs experimentally infected with EBOV, NP-specific antibody titers decreased within 28 days after infection, but neutralizing antibodies seemed to persist longer (*11*). Of note, Marburg virus IgG in convalescent *Rousettus aegyptiacus* bats decreased to undetectable titers at 3 weeks after infection (*12*). Nonetheless, the recent report of EBOV neutralizing antibodies in human survivors up to 40 years after infection (*13*) suggests a rather long-lasting but host-dependent antibody response after infection.

Reactivity of dog serum to EBOV-NP in ELISA and Western blots suggests exposure of the dogs to antigenically related ebolaviruses or Ebola-like viruses, as previously described for pigs (*5*). In our study, a novel ebolavirus, referred to as Bombali virus, which was recently discovered in insectivorous bats from the Bombali District in Sierra Leone (*3*), may account for cross-reactivity of the dog serum to EBOV-NP. The virus neutralization induced by specific binding to the EBOV surface glycoprotein suggests exposure of the dogs to EBOV or to a closely related ebolavirus eliciting cross-neutralizing antibodies. Although in vitro assays using an EBOV glycoprotein-pseudotyped virus revealed that infectivity is restricted in canine cells (*14*), detection of EBOV (cross-)neutralizing antibodies in dogs supports susceptibility to natural EBOV or ebolavirus infection.

The dog with the highest neutralizing titer (1:45) was 48 months of age; other dogs with neutralizing antibodies were 28–72 months of age at the time of blood collection, suggesting exposure during the West Africa EVD outbreak. However, information on past clinical signs in the dogs was not recorded, and the route of exposure or potential infection remains unknown. Exposure of dogs during the EVD outbreak in Gabon was assumed to result from consuming virus-infected carcasses or licking vomitus from EVD patients (*8*). Samples from those dogs, which displayed no clinical signs, tested negative for EBOV RNA (*8*). Furthermore, recent testing of 240 swab samples from dogs from Bombali District revealed no detectable filovirus RNA in the specimens; serologic assays were not performed (*3*).

Although most seropositive dogs in our study were potentially exposed to the virus during the EVD epidemic, 2 dogs with neutralizing antibodies (titers 1:16 and 1:27) were only 16 and 18 months of age, indicating contact with ebolavirus after the World Health Organization officially declared the end of the EVD outbreak in Sierra Leone by mid-March 2016 (*15*). Of note, some of the seropositive dog samples from Gabon were collected from areas without reported human EVD cases (*8*). These findings suggest exposure and immunogenic stimulation of free-ranging dogs by a source other than secretions from acutely infected patients or infection with a heterologous ebolavirus circulating in wildlife reservoir hosts.

To date, neither evidence of clinical EVD in dogs nor virus shedding with subsequent transmission to humans has been reported. However, whether dogs play an active role in EBOV ecology, represent dead-end hosts, or act as passive virus carriers mechanically spreading the virus after licking and feeding on infected carcasses or fomites remains unknown. Therefore, organ tissues (including salivary glands, bladder, and intestines) or secretions that might lead to virus shedding and transmission should be collected from dogs during any future EVD epidemic.

This report of EBOV neutralizing antibodies in dogs suggests their susceptibility to natural infection by EBOV or antigenically related ebolaviruses. Considering the abundance of dogs and their close association with humans in Africa, the comparably low number of human EVD outbreaks in the past most likely indicates that dogs do not represent a reservoir or intermediate host for EBOV.

Appendix Additional methods and limitations with regard to study of Ebola virus neutralizing antibodies in dogs from Sierra Leone, 2017.
