# Supplementary material for: Ebola Virus Neutralizing Antibodies in Dogs from Sierra Leone, 2017
Source: Emerg Infect Dis. 2020 Apr;26(4):760–3. doi: 10.3201/eid2604.190802 (PMC7101121; doi:10.3201/eid2604.190802)
Supplement: Appendix — Additional methods and limitations with regard to study of Ebola virus neutralizing antibodies in dogs from Sierra Leone, 2017. [file 19-0802-Techapp-s1.pdf]

Article DOI: https://doi.org/10.3201/eid2604.190802

Ebola Virus Neutralizing Antibodies in Dogs from Sierra Leone, 2017

Appendix

Methods

Animal sampling

We sampled 300 owned and apparently healthy dogs from Moyamba district from different communities (Figure 1, Table 2) between September and November 2017. Communities such as Moyamba Junction and Taiama have been known for their history of EVD patients during the EVD epidemic (2014-2016). Dogs are free ranging within the communities. Owners do not feed their dogs. Thus, dogs scavenge for food within the communities. Alternatively, dogs go hunting for food in the nearby forests. During the EVD outbreak, unsafe burials were performed allowing the dogs to come into contact with these bodies as a possible source of infection.

Most of the sampling areas / communities are close to major bat caves. Therefore, dogs may be exposed to bats directly or indirectly.

Control serum samples

For the establishment of the dog ELISA, we used 72 sera collected previously from German dogs as negative controls. These samples were also used to determine the cut-off by calculating the mean plus 3 standard deviations. None of the German sera ranged above that cut-off. Defined positive controls were unfortunately not available prior to our testing. Thus, we used sera from VLP immunized pigs as a positive control in ELISA. For Western Blot, His-tag specific monoclonal antibodies were used to detect the antigen.

Indirect IgG ELISA based on *E. coli* derived EBOV NP

The ELISA was performed as described (*1*) with the modification of using a horseradish peroxidase-labelled Protein A/G-specific conjugate.

Virus neutralization test (VNT) using authentic EBOV

Sera were incubated at 56°C for 30 min for complement inactivation. Then, sera were serially diluted and 100 $TCID_{50}$ units of Ebola virus (Zaire, isolate Mayinga, AF086833) were added to each serum dilution. Following incubation at 37°C for 1 hour, Vero cell suspension in DMEM containing 2% fetal calf serum was added. Plates were then incubated at 37°C with 5% CO_2 and cytopathic effects (CPE) were analysed at 7 dpi. Neutralization was defined as reduction of CPE in serum dilutions compared to positive controls. For each sample, we performed four replicates. Neutralization titres were calculated as geometric mean titres (GMT) of these four replicates. We defined a sample negative if the serum sample did not exhibit any neutralization effect at a 1:8 dilution (the lowest dilution tested). A corresponding serum control was assayed for each sample to exclude any potential cytotoxic effects. As a positive control for neutralization, we routinely use a purified ovine anti EBOV IgG product that exhibits high neutralization titers (EBOTab) (*2*). Further controls included virus-infected cells without the addition of antisera to determine the cytopathic effect as well as non-infected cells as Mock control. All these controls performed as expected.

In addition to analysis of ELISA reactive dog sera in VNT, we tested a number of ELISA negative dog serum samples that turned out to be non-neutralizing and not toxic to the cell monolayers.

Further information concerning the trVLP-based VNT are mentioned elsewhere (*1*).

Limitations of the study

To our knowledge, no study has previously reported EBOV neutralizing antibodies in dogs from Sierra Leone. Nonetheless, there are some limitations of this study. The development of assays for dog surveillance is a strength of this study, however, a defined positive control serum panel is lacking. Further, in this study, we only test for IgG antibodies. Expanding future testing for IgM antibodies as well as testing for viral genome presence would broaden our knowledge of the role of dogs in the ebolavirus transmission chain. Another limitation is that the age of the animals is reported according to the dog owner which may not be absolutely accurate.

References

1. Fischer K, Jabaty J, Suluku R, Strecker T, Groseth A, Fehling SK, et al. Serological Evidence for the circulation of Ebolaviruses in pigs From Sierra Leone. J Infect Dis. 2018 218(suppl_5):S305–S11.

2. Dowall SD, Callan J, Zeltina A, Al-Abdulla I, Strecker T, Fehling SK, et al. Development of a cost-effective ovine polyclonal antibody-based product, EBOTAb, to treat ebola virus infection. J Infect Dis. 2016;213:1124–33. PubMed https://doi.org/10.1093/infdis/jiv565
